# Regulation of Interleukin-36γ/IL-36R Signaling Axis by PIN1 in Epithelial Cell Transformation and Breast Tumorigenesis

**DOI:** 10.3390/cancers14153654

**Published:** 2022-07-27

**Authors:** Muna Poudel, Poshan Yugal Bhattarai, Pratikshya Shrestha, Hong Seok Choi

**Affiliations:** College of Pharmacy, Chosun University, 309 Pilmun-daero, Gwangju 61452, Korea; muna@chosun.kr (M.P.); poshanb@chosun.kr (P.Y.B.); rabbu_pratikshya@chosun.kr (P.S.)

**Keywords:** interleukins, activator protein 1, peptidyl-prolyl cis-trans isomerase NIMA-interacting 1

## Abstract

**Simple Summary:**

Members of the interleukin (IL)-1 cytokine family exhibit dual functions in the regulation of inflammation and cancer. Recent studies have shown the critical role of IL-36γ, the newly identified IL-1 family member, in the regulation of cellular processes implicated in the progression of cancer. Therefore, the underlying mechanism of IL-36γ in tumor development is of considerable interest. Here, we identified the pivotal role of IL-36γ in the proliferation of breast cancer cells. Consistently, IL-36γ was found to promote epithelial cell transformation via the activation of c-Fos, c-Jun, and AP-1 transcription factors, followed by the IL36R-mediated MEK/ERK and JNK/c-Jun cascades. Furthermore, our findings demonstrate the critical role of PIN1 in the regulation of IL-36γ-induced mammary gland tumorigenesis.

**Abstract:**

Given the increasing recognition of the relationship between IL-1 cytokines, inflammation, and cancer, the significance of distinct members of the IL-1 cytokine family in the etiology of cancer has been widely researched. In the present study, we investigated the underlying mechanism of the IL-36γ/IL-36R axis during breast cancer progression, which has not yet been elucidated. Initially, we determined the effects of IL-36γ on the proliferation and epithelial cell transformation of JB6 Cl41 mouse epidermal and MCF7 human breast cancer cells using BrdU incorporation and anchorage-independent growth assays. We found that treatment with IL-36γ increased the proliferation and colony formation of JB6 Cl41 and MCF7 cells. Analysis of the mechanism underlying the neoplastic cell transformation revealed that IL-36γ induced IL-36R-mediated phosphorylation of MEK1/2, ERK1/2, JNK1/2, and c-Jun, resulting in increased c-Fos, c-Jun, and AP-1 activities in JB6 Cl41 and MCF7 cells. Furthermore, the IL-36γ-induced tumorigenic capacity of MCF7 cells was considerably enhanced by PIN1, following MEK/ERK and JNK/c-Jun signaling. Interestingly, blocking PIN1 activity using juglone suppressed the IL-36γ-induced increase in the anchorage-independent growth of 4T1 metastatic mouse breast cancer cells. Finally, in a syngeneic mouse model, IL-36γ-induced tumor growth in the breast mammary gland was significantly inhibited following PIN1 knockout.

## 1. Introduction

Breast cancer is the most prevalent cause of cancer-related death among women worldwide [1]. The molecular mechanisms involved in breast tumorigenesis, including tumor initiation, promotion, angiogenesis, and metastasis, are closely related to inflammatory processes [2]. Cytokines, including interleukins (ILs), chemokines, interferons, and tumor necrosis factors, are central mediators of inflammation in the tumor microenvironment that affect the development of breast tumors [3,4,5,6]. Among the various cytokines, the role of ILs belonging to the IL-1 family is particularly interesting in cancer, as they display both pro- and anti-tumorigenic properties, depending on the cancer type [7]. In previous reports, ILs such as IL-18, IL-33, and IL-1α were found to have pro-tumorigenic effects in pancreatic cancer, breast cancer, and leukemia [8,9,10]. In contrast, IL-37 and IL-38 have been reported to exert tumor-suppressive properties in lung and colorectal cancer [11,12]. However, the role of the newly discovered members of the IL-1 cytokine family, including IL-36, in breast cancer is poorly understood. 

IL-36 is a subset of the IL-1 cytokine family with pro-inflammatory effects [13], and includes four members: IL-36α, IL-36β, IL-36γ, and IL-36 receptor antagonists (formerly known as IL-1F6, IL-1F8, IL-1F9, and IL-1F5) [14]. Members of the IL-36 family are expressed in a wide range of cell types, with monocytes and epithelial cells expressing the highest levels [15,16,17]. IL-36 isoforms signal via the IL-36 receptor (IL-36R), also known as IL-1 receptor-like 2 (IL-1RL2) or IL-1 receptor-related protein 2 (IL-1Rrp2) [18]. IL-36 binds to IL-36R and activates the nuclear factor-kappa B (NF-κB) and mitogen-activated protein kinase (MAPK) signaling pathways, which are involved in various cellular processes, such as proliferation, differentiation, transformation, invasion, and apoptosis [18,19,20]. Initial studies have reported a pivotal role of IL-36 in the initiation of inflammation-related diseases. For example, IL-36α and IL-36γ showed pro-inflammatory effects by promoting the expression of chemokines in colonic epithelial cells, suggesting their role in the pathogenesis of gut inflammation [21]. Serum levels of IL-36β, which exerts pro-inflammatory effects, are elevated in patients with rheumatoid arthritis [22]. In a mouse model of psoriasis, IL-36α, IL-36β, and IL-36γ levels were markedly increased, suggesting their role in skin inflammation [23]. Interestingly, IL-36γ exhibits both tumor-promoting and -suppressing properties in several cancers. For example, IL-36γ inhibits lung metastasis and melanoma tumor growth by inducing the activation and proliferation of CD8+ and natural killer (NK) cells [24]. In contrast, IL-36γ significantly increased colorectal cancer (CRC) cell proliferation [25]. In addition, the knockout (KO) of IL-36γ reduced tumors in the small intestine of mice, indicating a pro-tumorigenic role of IL-36γ in the progression of colon cancer [26]. Although the dual function of IL-36γ in cancers has been demonstrated in these studies, its underlying molecular mechanism in the development of breast cancer has not yet been elucidated. 

Peptidyl-prolyl cis-trans isomerase NIMA-interacting 1 (PIN1), a peptidyl-prolyl cis/trans isomerase (PPIase), regulates proteins with phosphorylated proline-directed serine or threonine motifs, which mediate an essential signaling mechanism in cell proliferation and tumorigenesis [27,28]. The role of PIN1 in tumor-promoting inflammation has been extensively studied. In a mouse model, the KO of PIN1 attenuated ulcerative colitis induced by dextran sodium sulfate [29]. PIN1 has been shown to regulate the production of pro-inflammatory cytokines such as IL-1β and IL-18 in macrophages [30]. Furthermore, PIN1 enhances IL-34-induced breast cancer development via activation of the MEK/ERK and JNK/c-Jun signaling pathways [31]. In a previous report, PIN1 was shown to bind to MEK and c-Jun, thus mediating the MAPK signaling pathway during breast tumor development [32,33]. In addition, juglone, a PIN1 inhibitor, suppressed tumor growth of breast cancer cells by inhibiting the activity of PIN1 in syngeneic models in vivo [31]. Although the function of PIN1 has been studied extensively, it is unclear whether PIN1 participates in the IL-36γ signaling pathway during breast cancer development. 

To the best of our knowledge, this is the first study to demonstrate that the IL-36γ/IL36R signaling axis plays a pivotal role in breast carcinogenesis. IL-36γ activates the MEK/ERK and JNK/c-Jun signaling axis via IL-36R in breast cancer, which eventually results in increased transcriptional activity of c-Fos, c-Jun, and AP-1. In addition, PIN1 enhanced neoplastic cellular transformation and in vivo growth of breast tumors induced by IL-36γ. Our study highlights the pro-tumorigenic effects of IL-36γ and the regulatory role of PIN1 in IL-36γ-induced breast tumorigenesis. 

## 2. Materials and Methods

### 2.1. Reagents and Antibodies

Recombinant mouse and human IL-36γ were obtained from R&D Systems (Minneapolis, MN, USA). Anti-IL36R was obtained from Abcam (Cambridge, MA, USA). The PIN1 inhibitor juglone (5-hydroxy-1,4-naphthoquinone), was obtained from Sigma-Aldrich (St. Louis, MO, USA). PD98059 and SP600125 were purchased from Calbiochem-Novabiochem (San Diego, CA, USA). Eagle’s minimal essential medium, L-glutamine, gentamicin, and fetal bovine serum (FBS) were purchased from Invitrogen (Carlsbad, CA, USA). Cell proliferation enzyme-linked immunosorbent assay (ELISA) and Bromodeoxyuridine/5-bromo-2′-deoxyuridine (BrdU) (colorimetric assay) were purchased from Roche Applied Science (Indianapolis, IN, USA). A Dual-Luciferase Reporter Assay Kit was purchased from Promega (Madison, WI, USA). The antibodies acquired from Cell Signaling Technology (Danvers, MA, USA) were as follows: MEK1/2, p44/42 MAPK (ERK1/2), SAPK/JNK, c-Fos, phospho-MEK1/2, phospho-p44/42 MAPK (ERK1/2), phospho-SAPK/JNK, phospho-c-Fos, and phospho-c-Jun. The antibodies against PIN1, and c-Jun were obtained from Santa Cruz Biotechnology (Dallas, TX, USA). Antibody against XPRESS was acquired from Invitrogen. Anti-β-actin antibody was obtained from Sigma-Aldrich. 

### 2.2. Cell Culture and TRANSFECTION

JB6 Cl41, MCF7, and 4T1 cells were cultured and maintained at 37 °C in a humidified atmosphere containing 5% CO_2_ as mentioned previously [31]. JetPEI was purchased from Polyplus-transfection (Illkrich, France). Lipofectamine^™^ 3000 transfection reagent was obtained from Invitrogen.

### 2.3. CRISPR/Cas9 KO System 

To KO genes using the CRISPR/Cas9 system, guide RNAs targeting human *IL36R* (1st, 5′-CACCGAACTTACTTTATAACACAT-3′; 2nd, 5′-CACCGTAACGAGATTAAAGGGGAG), mouse *IL36R* (1st, 5′-CACCGTTGTGAAGAGATTAAAGCG-3′; 2nd, 5′-CACCGTACCGAATTTGATCCCTCA-3′), mouse *PIN1* (5′-GATGAGCGGGCCCGTGTTCA-3′) and human *PIN1* (1st, 5′-GATGAGCGGGCCCGTGTTCA-3′; and 2nd 5′-GAAGATCACCCGGACCAAGG-3′) were designed using the Broad Institute CRISPick portal (https://portals.broadinstitute.org/gppx/crispick/public, accessed on 6 July 2021) and cloned into the BbsI site of the eSpCas9(1.1)-T2A-puro vector (a gift from Andrea Nemeth, Addgene plasmid #101039). At 48 h following transfection, the cells were harvested and used for the experiment without single-cell isolation.

### 2.4. Cell Proliferation Assay Using 5-bromo-2′-deoxyuridine (BrdU) Incorporation

JB6 Cl41, MCF7, and 4T1 cells were seeded (5 × 10^3^ cells/well) in 96-well plates in 100 µL of MEM, DMEM, or RPMI supplemented with 5% or 10% FBS, as appropriate. After 24 h, the cells were treated with or without IL-36γ for 48 h, labeled with 10 µL/well BrdU labeling solution, and then incubated for 4 h at 37 °C in a 5% CO_2_ atmosphere. Cell proliferation was determined by measuring the absorbance at 370 nm.

### 2.5. Immunoblot Analysis

Cells were lysed in radioimmunoprecipitation assay lysis buffer. Proteins were resolved by sodium dodecyl sulfate-polyacrylamide gel electrophoresis and blotted onto polyvinylidene difluoride membranes. The membranes were blocked in 5% skim milk and incubated with the indicated primary antibody overnight at 4 °C. After incubation with horseradish peroxidase (HRP)-conjugated secondary antibodies from rabbits or mice, the protein bands were detected using a chemiluminescence detection kit (HRP Chemiluminescent Substrate, Amersham Biosciences, Piscataway, NJ, USA) in an LAS4000 system (GE Healthcare Biosciences, Pittsburgh, PA, USA).

### 2.6. Anchorage-Independent Cellular Transformation Assay (Soft Agar Assay)

The effect of IL-36γ on cellular transformation was investigated in the JB6 Cl41, MCF7, and 4T1 cells. Briefly, 8 × 10^3^ cells were treated with different concentrations of IL-36γ in 1 mL of 0.3% basal Eagle’s medium containing 10% FBS, 2 mM L-glutamine, and 25 µg/mL gentamicin. The cultures were maintained at 37 °C in a 5% CO_2_ incubator for 14–18 d. The resultant cell colonies were scored using an Axiovert 200M fluorescence microscope and AxioVision software (both from Carl Zeiss, Thornwood, NY, USA). Six images were obtained for each group. Colony size and number were measured using ImageJ software (NIH, Bethesda, MD, USA).

### 2.7. Luciferase Assay

JB6 Cl41 and MCF7 cells transfected with AP-1-, c-Jun-, and c-Fos-Luc were lysed in passive cell lysis buffer (Promega). The firefly luciferase activity in cell lysate was measured using the GloMax^®^-Multi Detection System (Promega). The Renilla luciferase activity generated by pRL-TK-luciferase plasmid (Promega) was used to normalize the transfection efficiency. c-Fos-Luc (pFos-WT GL3) and c-Jun-Luc (JC6GL3) promoter constructs were a gift from Dr. Ron Prywes (Columbia University, NY, USA). AP-1 luciferase reporter plasmid (−73/+63 collagenase-luciferase) was kindly provided by Dr. Dong Zigang (Hormel Institute, University of Minnesota, Austin, MN, USA).

### 2.8. Tumorigenicity Assay in BALB/c Mice

Six-week-old female BALB/c mice (18–20 g) were obtained from Samtako Co. (Osan, Republic of Korea), acclimatized for one week, and maintained in a clean room at the College of Pharmacy, Chosun University (Gwangju, Korea). The animals were caged under filtered pathogen-free air at a temperature between 20 and 23 °C, with a 12 h/12 h light/dark cycle and relative humidity of 50%. The animals were fed commercial rat chow (Orient Bio, Co., Seongnam, Korea). The animal study protocols were approved by the Animal Care Committee of Chosun University. Mice were randomly divided into four groups of six animals each. Mouse breast cancer 4T1 cells transfected with sgCtrl or sgPIN1 were then implanted into the mammary glands of the mice either in the presence or absence of IL-36γ and allowed to grow for 14 days. The mice were observed daily for tumor growth. Tumor volume was calculated using the formula: V = (ab^2^)/2, where ‘a’ represents the longest diameter and ‘b’ represents the shortest diameter of the tumor.

### 2.9. Statistical Analysis 

Statistical analyses were performed using Prism 8 software (GraphPad Software, San Diego, CA, USA). One-way analysis of variance (ANOVA) followed by Tukey’s multiple comparisons test were used for multiple comparison analyses. *p* < 0.05 was considered statistically significant.

## 3. Results

### 3.1. IL-36γ Induced Proliferation and Anchorage-Independent Growth of JB6 Cl41 and MCF7 Cells

**Figure 1:**
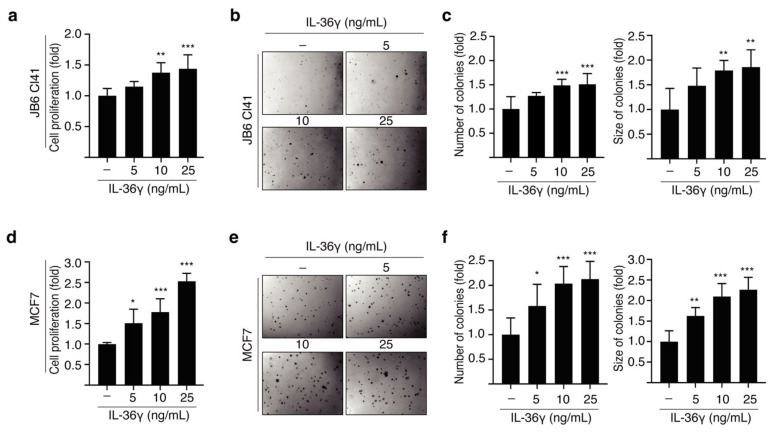
Effects of IL-36γ on anchorage-independent growth and epithelial cell transformation in vitro. (**a**) JB6 Cl41 cells were treated with the indicated concentrations of IL-36γ for 48 h, and cell proliferation was determined using the BrdU incorporation assay. (**b**,**c**) JB6 Cl41 cells were treated with different concentrations of IL-36γ as indicated and grown in a soft agar matrix by incubation at 37 °C in a 5% CO_2_ atmosphere for 14 days. Representative images from three separate experiments are shown (**b**), followed by a calculation of the average colony numbers and sizes (diameter > 80 μm) (**c**). (**d**) MCF7 cells were treated with various concentrations of IL-36γ for 48 h, and cell proliferation was determined using the BrdU incorporation assay. (**e**,**f**) MCF7 cells were treated with the indicated concentrations of IL-36γ and grown in a soft agar matrix by incubation at 37 °C in a 5% CO_2_ atmosphere for 14 days. Representative images from three separate experiments are shown (**e**), followed by a calculation of the average colony numbers and sizes (diameter > 100 μm) (**f**). Error bars represent the mean ± standard deviation (S.D.) of triplicate measurements from at least three individual experiments. Statistical analyses were performed using one-way ANOVA (* *p* < 0.05, ** *p* < 0.01, *** *p* < 0.001, compared to the control groups). In b and e, magnification, 100×.

JB6 Cl41 cells proliferate in an anchorage-independent manner following treatment with tumor promoters [31]. To study whether IL-36γ affects the proliferation and transformation of JB6 Cl41 cells, we performed BrdU incorporation and soft agar assays. IL-36γ stimulation significantly increased the proliferation of JB6 Cl41 cells in a dose-dependent manner (Figure 1a). Consistent with the increase in cell proliferation, the colony formation of JB6 Cl41 cells was also increased following treatment with IL-36γ (Figure 1b,c). To investigate the effect of IL-36γ on the growth of MCF7 breast cancer cells, we initially performed a BrdU incorporation assay. We observed a notable increase in the number of MCF7 cells following treatment with IL-36γ (Figure 1d). Similarly, treatment with IL-36γ significantly increased the number and size of colonies of MCF7 cells in a dose-dependent manner (Figure 1e,f). Together, our results indicate that IL-36γ exerts pro-tumorigenic effects resulting from increased cell proliferation and transformation.

### 3.2. IL-36γ Induced MEK/ERK and JNK/c-Jun Signaling Pathways via IL36R in JB6 Cl41 Cells

**Figure 2:**
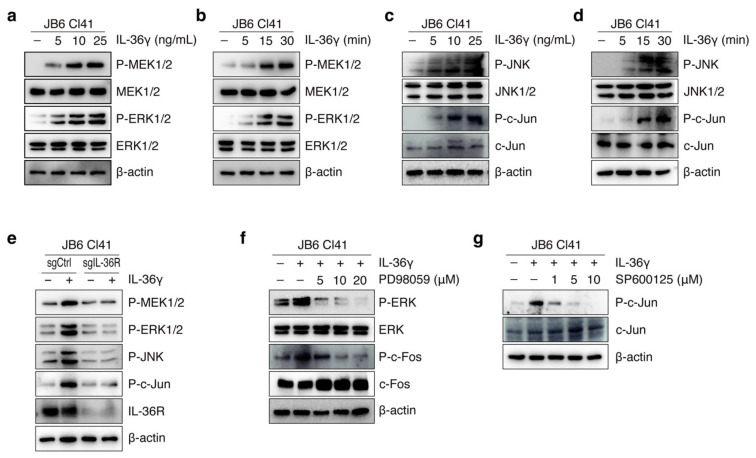
Effects of IL-36γ on the MEK/ERK and JNK/c-Jun signaling pathway in JB6 Cl41 cells. (**a**–**d**) Cells were treated with the indicated concentrations of IL-36γ for 30 min (**a**,**c**) or 10 ng/mL IL-36γ for the indicated times (**b**,**d**). Proteins in the whole-cell lysates were analyzed by Western blotting. (**e**) Cells were transfected with mouse sgCtrl and sgIL-36R. After 48 h of transfection, cells were serum-starved for 24 h prior to treatment with 10 ng/mL of IL-36γ. After 15 min, total protein was collected, and the levels of the indicated protein were determined by Western blotting using specific antibodies. (**f**,**g**) Cells were pre-treated with the indicated concentrations of PD98059 (**f**) and SP600125 (**g**) for 12 h and then treated with 10 ng/mL IL-36γ for 15 min. Proteins in the whole-cell lysates were analyzed by Western blotting. (**a**–**g**) Representative blots are shown from at least three independent experiments. 3.3. PIN1 Enhances AP-1 Activity and IL-36γ-Induced Transformation of JB6 Cl41 Cells. The full uncropped blots for Figure 2 can be found in Figure S1.

IL-36R has previously been reported to trigger the MAPK signaling cascade, which is primarily involved in the regulation of cancer cell growth and tumorigenesis [31,34]. As IL-36γ is known to be a ligand of IL-36R [18], we assessed the effect of IL-36γ on MEK/ERK and JNK/c-Jun signaling. We found that IL-36γ activated the MAPK pathway in JB6 Cl41 cells as evidenced by a dose- and time-dependent increase in the phosphorylation levels of MEK1/2 and ERK1/2 (Figure 2a,b) together with JNK1/2 and c-Jun (Figure 2c,d). To further determine whether IL-36γ-triggered MEK/ERK and JNK/c-Jun cascades were mediated via IL36R, we transfected mouse sgCtrl and sgIL-36R into JB6 Cl41 cells and subsequently treated them with IL-36γ. The inducing effects of IL-36γ on MEK1/2, ERK1/2, JNK1/2, and c-Jun phosphorylation were reduced in IL-36R KO cells as compared to that in control cells (Figure 2e). Similarly, PD98059, an inhibitor of MEK1/2, and SP600125, a JNK1/2 inhibitor, suppressed the phosphorylation of ERK1/2, c-Fos, and c-Jun (Figure 2f,g) which was increased by IL-36γ. Together, these results indicate that IL-36γ activates the MEK/ERK and JNK/c-Jun signaling pathways through IL-36R in JB6 Cl41 cells.

**Figure 3:**
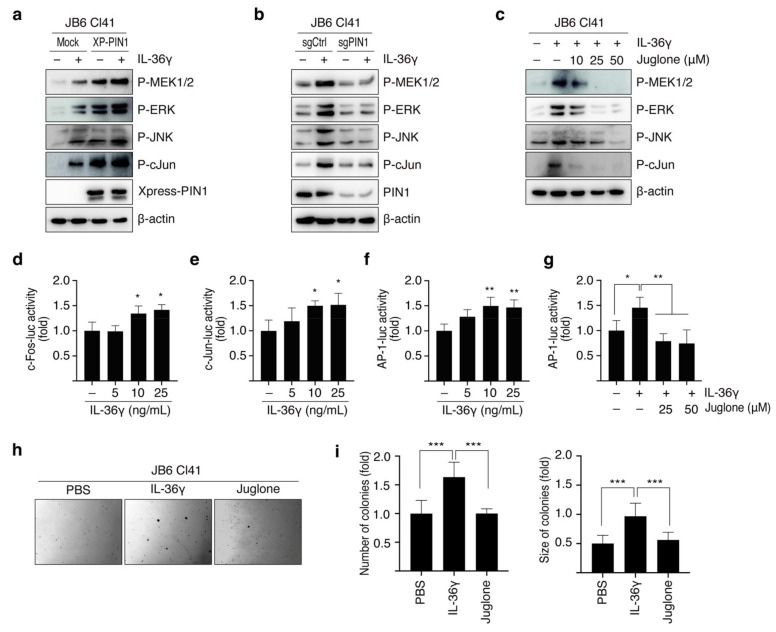
Effects of juglone in IL-36γ-induced AP-1 activity and cellular transformation of JB6 Cl41 cells. (**a**,**b**) Cells were transfected with mock and XP-PIN1 (**a**) or mouse sgCtrl and sgPIN1 (**b**), respectively. After 48 h of transfection, cells were serum-starved for 24 h and treated with 10 ng/mL IL-36γ for 15 min. Proteins in the whole-cell lysates were determined by Western blotting. (**c**) Cells were exposed to the indicated amount of juglone for 12 h, prior to 15 min of treatment with 10 ng/mL IL-36γ. The levels of the indicated proteins were determined by Western blotting using specific antibodies. (**d**–**f**) The luciferase reporters c-Fos-Luc (**d**), c-Jun-Luc (**e**), and AP-1-Luc (**f**) were co-transfected along with the pRL-TK vector into cells. Cells were serum-starved for 24 h, following 24 h of transfection prior to dose-dependent treatment with IL-36γ for 24 h. (**g**) The luciferase reporter AP-1-Luc was co-transfected with pRL-TK vector into cells. Cells were pre-treated with the indicated doses of juglone, followed by 24 h of serum starvation and then exposed to 10 ng/mL IL-36γ for 24 h. (**h**,**i**) Cells were treated with 10 ng/mL IL-36γ either alone or in combination with 50 μM juglone in a soft agar matrix and incubated at 37 °C in a 5% CO_2_ atmosphere for 14 days. Representative pictures of the colonies from three separate experiments (**h**) and the quantification of the colony sizes and numbers (**i**). Magnification, 100×. Error bars indicate the mean ± S.D. of triplicate measurements. Statistical analyses were performed using one-way ANOVA (* *p* < 0.05, ** *p* < 0.01, *** *p* < 0.001, compared to the control group or only-IL-36γ-treated group). The full uncropped blots for Figure 3 can be found in Figure S2.

PIN1 has been shown to interact with MAPK signaling components such as MEK1 [32] and c-Jun [33]. Therefore, we hypothesized that PIN1 may influence the IL-36γ-induced MEK/ERK and JNK/c-Jun signaling pathways. JB6 Cl41 cells were first transfected with Mock and XP-PIN1 or sgCtrl and sgPIN1, and then treated with IL-36γ. PIN1 overexpression enhanced IL-36γ-induced phosphorylation of MEK1/2, ERK1/2, JNK1/2, and c-Jun (Figure 3a). Conversely, PIN1 KO in JB6 Cl41 cells attenuated the IL-36γ-induced MEK1/2, ERK1/2, JNK1/2, and c-Jun phosphorylated levels (Figure 3b). Similarly, to further validate the regulatory function of PIN1 in the IL-36γ-induced MEK/ERK and JNK/c-Jun cascades, we blocked PIN1 activity using its inhibitor juglone in JB6Cl41 cells. Treatment with juglone decreased IL-36γ-induced MEK1/2, ERK1/2, JNK1/2, and c-Jun phosphorylation (Figure 3c). Furthermore, the roles of IL-36γ in the transcriptional activity of c-Fos, c-Jun, and AP-1 were investigated. IL-36γ markedly elevated the transcriptional activity of c-Fos and c-Jun (Figure 3d,e). Similarly, the transactivation of AP-1 was upregulated following IL-36γ treatment (Figure 3f). To better understand the role of PIN1 in IL-36γ-induced AP-1 activity and epithelial cell transformation, we first analyzed the effect of juglone on IL-36γ-induced AP-1 activity. We found that treatment with juglone markedly inhibited IL-36γ-induced transactivation of AP-1 (Figure 3g). Moreover, juglone dose-dependently inhibited IL-36γ-induced anchorage-independent growth of JB6 Cl41 cells (Figure 3h,i). Taken together, these findings demonstrate that PIN1 regulates MEK/ERK and JNK/c-Jun signaling stimulated by IL-36γ in JB6 Cl41 cells.

### 3.3. PIN1 Regulates IL-36γ-Induced MEK/ERK and JNK/c-Jun Signaling Pathways in MCF7 Cells

**Figure 4:**
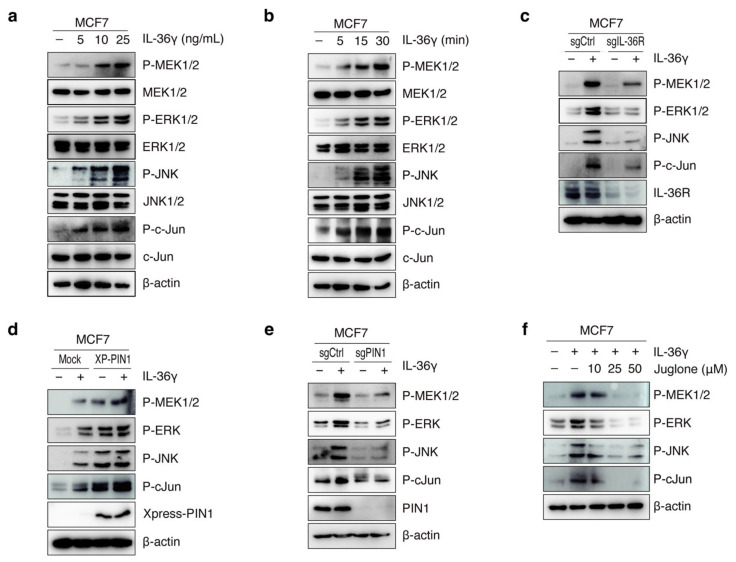
Role of PIN1 in the IL-36γ-induced MEK/ERK and JNK/c-Jun signaling pathways in MCF7 cells. (**a**,**b**) Cells were treated with the indicated concentration of IL-36γ for 30 min (**a**) or treated with 10 ng/mL IL-36γ for the indicated time (**b**). (**c**) Cells were transfected with human sgCtrl and sgIL-36R. After 48 h, cells were serum-starved for 24 h prior to treatment with 10 ng/mL IL-36γ. After 15 min, cells were harvested and lysed. Proteins in the whole-cell lysate were determined by Western blotting. (**d**,**e**) Cells were transfected with mock and XP-PIN1 (**d**) or sgCtrl and sgPIN1 (**e**), respectively. At 48 h after transfection, cells were serum-starved for 24 h and treated with 10 ng/mL IL-36γ for 15 min. (**f**) Cells were pre-treated with the indicated doses of juglone for 12 h prior to treatment with 10 ng/mL IL-36γ. After 15 min, cells were harvested and lysed. Proteins in the whole-cell lysates were analyzed by Western blotting. The blots shown are representative of at least three independent experiments.The full uncropped blots for Figure 4 can be found in Figure S3.

Given the role of IL-36γ in the activation of the MEK/ERK and JNK/c-Jun cascades, we further hypothesized that IL-36γ triggers the MAP signaling cascade in breast cancer MCF7 cells. Treatment with IL-36γ increased the phosphorylated forms of MEK1/2, ERK1/2, JNK1/2, and c-Jun in a dose- and time-dependent manner (Figure 4a,b). To examine the role of IL36R in IL-36γ-induced MEK/ERK and JNK/c-Jun signaling, cells were transfected with human sgCtrl and sgIL-36R followed by treatment with or without IL-36γ. Silencing of IL36R decreased the phosphorylation levels of MEK1/2, ERK1/2, JNK1/2, and c-Jun induced by IL-36γ in MCF7 cells (Figure 4c). To further evaluate the effects of PIN1 on the IL-36γ-induced MAPK pathway in breast cancer, we overexpressed and knocked out PIN1 in MCF7 cells, followed by IL-36γ treatment. As expected, PIN1 enhanced IL-36γ-induced phosphorylation of MEK1/2, ERK1/2, JNK1/2, and c-Jun (Figure 4d). Conversely, PIN1 KO significantly reduced IL-36γ-mediated induction of phosphorylated MEK1/2, ERK1/2, JNK1/2, and c-Jun in cells (Figure 4e). Furthermore, treatment with juglone inhibited the phosphorylation of MEK1/2, ERK1/2, JNK1/2, and c-Jun induced by IL-36γ (Figure 4f) in a dose-dependent manner. Collectively, these results demonstrate that PIN1 regulates IL-36γ-induced MEK/ERK and JNK/c-Jun signaling pathways in breast cancer.

### 3.4. PIN1 Promotes IL-36γ-Induced AP-1 Activity and Anchorage-Independent Growth of MCF7 Cells

**Figure 5:**
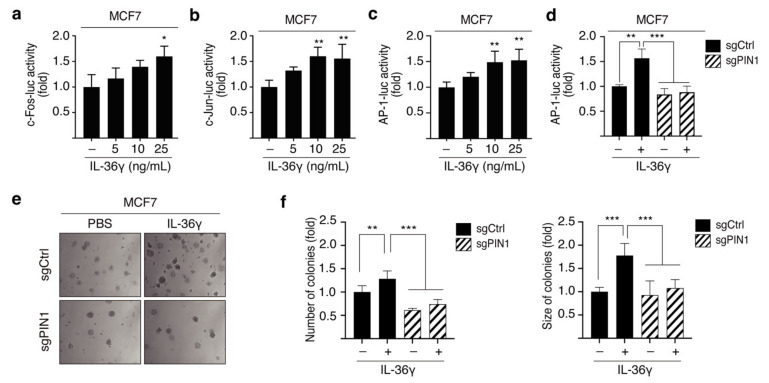
Role of PIN1 in IL-36γ-induced AP-1 activity and epithelial cell transformation. (**a**–**c**) The luciferase reporters c-Fos-Luc (**a**), c-Jun-Luc (**b**), and AP-1-Luc (**c**) were co-transfected with the pRL-TK vector into cells. Following 24 h of transfection, cells were serum-starved for 24 h prior to dose-dependent treatment with IL-36γ for 24 h. (**d**) The luciferase reporter AP-1-Luc and sgCtrl or AP-1-Luc and sgPIN1 were co-transfected into cells and incubated for 24 h. After being serum-starved for 24 h, cells were exposed to 10 ng/mL IL-36γ for another 24 h. (**e**) sgCtrl and sgPIN1 were transfected into cells and incubated for 48 h. Then, cells were treated with or without 10 ng/mL IL-36γ in a soft agar matrix and incubated at 37 °C in a 5% CO_2_ atmosphere for 14 days. Magnification, 100×. (**f**) Representative images from three separate experiments are presented (left), followed by a calculation of the average colony numbers and sizes (diameter > 200 μm, right). Error bars indicate the mean ± S.D. of triplicate measurements from three individual experiments. Statistical analyses were performed using one-way ANOVA (* *p* < 0.05, ** *p* < 0.01, *** *p* < 0.001, compared to the control group or only-IL-36γ-treated group).

To further investigate the role of IL-36γ in human breast cancer, we performed the luciferase reporter and soft agar assay in MCF7 cells. We initially evaluated the effects of IL-36γ on c-Fos and c-Jun promoter activity. The results showed that IL-36γ considerably raised the promoter activity of c-Fos (Figure 5a) and c-Jun (Figure 5b). Similarly, treatment with IL-36γ induced AP-1 transactivation in a dose-dependent manner (Figure 5c). Given the regulatory role of PIN1 in the IL-36γ-induced MAPK pathway, we next determined the effects of PIN1 on IL-36γ-induced AP-1 activity by transfecting sgCtrl and sgPIN1 into MCF7 cells, followed by treatment with IL-36γ. The results showed that the transactivation activity of AP-1 induced by IL-36γ was significantly decreased in sgPIN1-transfected MCF7 cells compared with sgCtrl-transfected MCF7 cells (Figure 5d). We further investigate the effects of PIN1 on IL-36γ-induced anchorage-independent growth of MCF7 cells. We found that the tumorigenic potential of MCF7 cells induced by IL-36γ was attenuated in PIN1 KO cells compared to that in control cells, as evident from the decreased formation of colonies (Figure 5e). Together, these results suggest that PIN1 regulates the IL-36γ-induced AP-1 transactivation to promote tumorigenesis in MCF7 cells. 

### 3.5. PIN1 Regulates IL-36γ-Induced 4T1 Cell Proliferation and Mammary Gland Tumorigenesis

**Figure 6:**
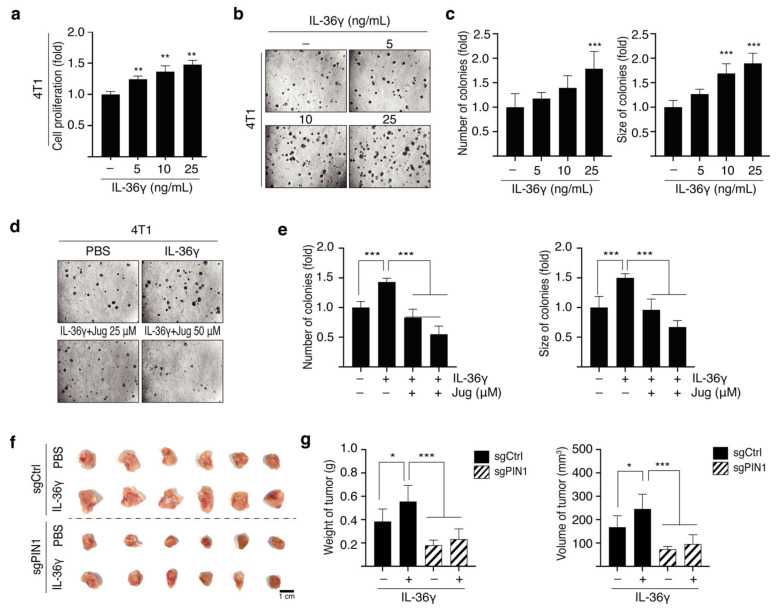
Role of PIN1 in IL-36γ-induced cell proliferation and mammary gland tumorigenesis. (**a**) 4T1 cells were seeded and treated with IL-36γ as indicated. Cell proliferation was estimated using the BrdU incorporation assay. (**b**,**c**) 4T1 cells were treated with the indicated doses of IL-36γ, subjected to a soft agar matrix, and incubated at 37 °C in a 5% CO_2_ atmosphere for 14 days. Representative images from three separate experiments are presented (**b**), followed by a calculation of the average colony numbers and sizes (diameter >200 μm) (**c**). (**d**,**e**) 4T1 cells were treated with the indicated concentrations of IL-36γ in the presence or absence of juglone, grown in a soft agar matrix, and incubated at 37 °C in a 5% CO_2_ atmosphere for 14 days. Representative images from three separate experiments are presented (**d**), followed by a calculation of the average colony numbers and sizes (diameter > 200 μm) (**e**). (**f**,**g**) WT 4T1 and PIN1 KO 4T1 cells were injected into the mammary gland of BALB/c mice in the presence or absence of 100 ng/mL IL-36γ and allowed to grow until tumors were formed. Shown are representative images of tumor (**f**), measured tumor weights, and tumor volumes (**g**). Error bars indicate the mean ± S.D. of triplicate measurements from three independent experiments. Statistical analyses were performed using one-way ANOVA (* *p* < 0.05, ** *p* < 0.01, *** *p* < 0.001, compared to the control group or only-IL-36γ-treated group, respectively). In b and d, magnification, 100×.

Given that IL-36γ induces tumorigenic potential in MCF7 breast cancer cells, we used a syngeneic mouse model to investigate the role of IL-36γ in promoting tumor development. Initially, we determined whether IL-36γ induces 4T1 cell proliferation. The results showed that 4T1 cell proliferation significantly and dose-dependently increased following treatment with IL-36γ (Figure 6a). In addition, IL-36γ markedly increased the anchorage-independent growth of 4T1 cells (Figure 6b,c). To understand the correlation between IL-36γ and PIN1 in the tumorigenic capability of 4T1 cells, we examined the effects of juglone, a PIN1 inhibitor, on the colony formation of 4T1 cells induced by IL-36γ. As expected, IL-36γ-induced colony formation was found to decrease following treatment with juglone (Figure 6d,e). Furthermore, the role of PIN1 in IL-36γ-induced breast tumor growth was studied in a BALB/c mouse allograft model. The results demonstrated that PIN1 ablation decreased IL-36γ-induced mammary gland tumor growth in mice (Figure 6f,g). Overall, these results demonstrate the regulatory role of PIN1 in IL-36γ-induced anchorage-independent growth of 4T1 cells and breast tumorigenesis. 

## 4. Discussion

Inflammation increases the risk of cancer and contributes to all stages of tumorigenesis, such as initiation, promotion, invasion, and metastasis [35]. Tumors develop at the sites of infection and chronic inflammation [36]. Cytokines have long been involved in inflammation-associated cancers [37]. IL-1 family members are largely known as critical cytokines that regulate inflammation and tumor progression [38]. IL-36γ, a recently identified member of the IL-1 cytokine family, plays a crucial role in chronic inflammatory diseases, such as asthma, dermatitis, chronic obstructive pulmonary disease, and psoriatic inflammation [39,40,41]. Despite the involvement of IL-36γ in inflammation, its role in cancer is still under investigation, with limited studies suggesting debatable effects. CB57/BL6 mice injected with B16-IL-36γ showed decreased melanoma tumor growth [24]. Similarly, 4T1-IL-36γ-injected CB57/BL6 mice exhibited increased Th1 anti-tumorigenic responses and decreased lung metastasis, indicating its anti-tumor effects [24]. However, increased levels of IL-36γ have been identified in non-small cell lung cancer and colon cancer tissues [26,42]. In addition, IL-36γ exerts pro-tumorigenic effects resulting from increased proliferation, migration, and invasion of colon cancer cells [25]. In this study, we present evidence that IL-36γ induces breast cancer cell proliferation in vitro. Furthermore, PIN1 enhances neoplastic cellular transformation and breast tumorigenesis in vitro and in vivo via IL-36γ/IL-36R signaling pathways. 

According to several studies, the enhancement of cellular proliferation is linked to IL-36R signaling, which has been observed in distinct cell types, including CD8+T, CD4+T, NK, and intestinal epithelial cells [25,43]. IL-36R signaling has previously been shown to be pathogenic for persistent intestinal inflammatory conditions, including inflammatory bowel disease and colitis [44,45]. In the lungs, inflammation was attenuated in IL-36R KO mice even after exposure to cigarette smoke, indicating that IL-36R is a critical component of the pro-inflammatory response [46]. Furthermore, tumor development was reduced in mice injected with IL-36R KO CT26 colon cancer cells [25]. IL-36R signaling is triggered by binding to its agonist (IL-36α, IL-36β, and IL-36γ) [18]. Specifically, IL-36α, IL-36β, and IL-36γ, members of the IL-1 cytokine family, stimulate the IL-36R-mediated MAPK and NF-κB signaling pathways, resulting in the induction of pro-inflammatory cytokines, such as IL-8, IFNγ, CXCL1, and CCL20 [18,47]. IL-36R agonists are differentially expressed in diseases ranging from inflammation to cancer. For instance, IL-36α and IL-36γ, but not IL-36β, are highly expressed in imiquimod-induced mouse skin inflammation and human psoriasis [48]. In addition, among the three IL-36 (α, β, and γ), macrophages overexpress only IL-36γ during lung inflammation following mycobacterial infection [49]. IL-36γ plays a pro-inflammatory role in the lungs of mice by promoting airway hyper-responsiveness and inducing NF-κB activity [50]. Interestingly, in the case of cancer, IL-36α, IL-36β, and IL-36γ showed distinct potencies in driving the proliferation of HT29 colon cancer cells, with IL-36γ showing the highest proliferative effect through the activation of the ERK and PI3K/AKT signaling pathways [25]. Despite its role in chronic inflammation and cancer growth, the underlying molecular mechanisms of IL-36γ during epithelial cell transformation and breast tumorigenesis have not yet been elucidated. Here, we identified a crucial role for IL-36γ in driving the proliferation of mouse epithelial JB6 Cl41 cells. Furthermore, IL-36γ strongly increased phosphorylation of MEK1/2, ERK1/2, JNK1/2, and c-Jun in JB6 Cl41 cells. In addition, the KO of IL-36R suppressed IL-36γ-induced phosphorylation of MEK1/2, ER1/2, JNK1/2, and c-Jun in JB6 Cl41 cells. Moreover, PD98059 and SP600125, inhibitors of MEK and JNK, respectively, potently attenuated the MEK/ERK and JNK/c-Jun signaling cascades induced by IL-36γ in JB6 Cl41 cells. These data suggest an essential role for IL-36γ/IL-36R in promoting tumorigenesis through activation of the MEK/ERK and JNK/c-Jun signaling pathways. 

The IL-1 cytokine family comprises critical molecules that regulate intracellular signaling cascades, such as p38 MAPK, ERK, NFkB, and JNK [9,51,52]. Such signaling pathways mediate fundamental biological phenomena, including cell proliferation, differentiation, apoptosis, transformation, and metastasis [19,53,54]. IL-18, a member of the IL-1 family of cytokines, increases the migration of MDA-MB-468, MDA-MB-231, and BT-549 human breast cancer cells through the p38 MAPK signaling pathway [55]. In gastric cancer, VEGF-enhanced production of IL-18 results in increased migration of human gastric cancer SNU-601 cells, followed by activation of the ERK signaling pathway [56]. ERK signaling has been shown to activate AP-1 transcription factor, which is implicated in tumorigenesis [31]. AP-1 is a dimeric transcription factor that contains members of the fos, jun, activating transcription factor (ATF), and musculoaponeurotic fibrosarcoma (MAF) protein families [57]. In our previous study, we found that AP-1 is an essential transcription factor in JB6 Cl41 epithelial cell transformation induced by IL-33, an IL-1 family cytokine. In addition, IL-33 induced AP-1 subunits, c-Fos, and c-Jun, resulting in breast tumor growth via MEK/ERK and JNK/c-Jun signaling [9]. IL-36γ activates the NF-κB and AP-1 (c-Jun) transcription factors for the induction of chemokines, which directs the recruitment of neutrophils to inflammatory sites and thus plays a crucial role in mucosal inflammation [58]. However, it is largely unknown whether AP-1 activity is regulated by IL-36γ/IL-36R signaling during tumor development. Our study provides the first direct evidence that IL-36γ increases the transcriptional activity of the AP-1 subunits c-Fos and c-Jun. Similarly, IL-36γ increased AP-1 transactivation activity in JB6 Cl41 cells. Furthermore, IL-36γ markedly induced anchorage-independent growth of JB6 Cl41 cells via the activation of AP-1 signaling. To the best of our knowledge, this study provides the first evidence regarding the role of IL-36γ/IL36R signaling in epithelial cell transformation. 

According to a recent study, IL-36γ exerts a critical function in the progression of colon cancer [26]. Knockout of IL-36γ led to decreased tumor incidence in various mice models of colon cancer, such as AOM/DSS, AOM/Vil-Cre, Trp53fl/fl, and ApcMin/+ [26]. Proliferation of CRC cell lines was also increased by IL-36R-mediated IL-36γ signaling [25]. In addition, cytokine-derived keratinocytes, regulatory T cells, intestinal epithelial cells, and colonic subepithelial myofibroblasts have all been found to proliferate in response to IL-36R activation [25,59]. However, the molecular mechanism of IL-36γ/IL36R in breast cancer remains to be explored. In the present study, IL-36γ stimulated MEK/ERK and JNK/c-Jun signaling via IL-36R in MCF7 cells. Consistently, KO of IL-36R or PD98059 and SP600125 administration inhibited the IL-36γ-induced phosphorylation of MAPK signaling components, including MEK1/2, ERK1/2, JNK, and c-Jun, in MCF7 cells. IL-36γ elevated the transcriptional activity of c-Fos and c-Jun, which resulted in increased AP-1 transactivation. In addition, IL-36γ promoted the proliferation and tumorigenic capability of human breast cancer MCF7 cells. Furthermore, IL-36γ induced proliferation and neoplastic transformation of 4T1 metastatic mouse breast cancer cells. In addition, IL-36γ increased the growth of tumors in the in vivo allograft mouse model. Thus, our study demonstrates the tumor-promoting effects of IL-36γ in breast cancer and suggests that the IL-36γ/IL-36R axis may be a potential therapeutic target for patients with cancer.

PIN1 regulates the outcome of proline (Pro)-directed phosphorylation of the serine/threonine (Ser/Thr) motif, which is an important signaling mechanism implicated in cell proliferation, growth, and transformation [28]. A link between PIN1 and IL-1 cytokine family members has been identified in patients with pancreatic cancer. Additionally, PIN1-induced IL-18 expression promotes pancreatic cancer cell proliferation and motility, whereas PIN1 knockdown inhibits the tumor-promoting effect of IL-18 [60]. Interleukin receptor-associated kinase M (IRAK-M), which is targeted by PIN1, is critical for type-2 immunity and allergic airway inflammation induced by IL-33, which regulates the secretion of pro-inflammatory cytokines, such as IL-17A and IL-22, during lung infection [61,62]. Interestingly, in our previous report, the PIN1 inhibitor juglone suppressed the IL-22-induced tumorigenic potential of human breast cancer MCF7 cells via MAPK signaling [63]. This evidence supports our hypothesis that PIN1 regulates IL-36γ-induced epithelial cell transformation and tumorigenesis in breast cancer. The results showed that PIN1 KO lowered MEK1/2, ERK1/2, JNK1/2, and c-Jun phosphorylation induced by IL-36γ in both JB6 Cl41 and MCF7 cells. Consistently, administration of the PIN1 inhibitor juglone resulted in a significant reduction in IL-36γ-induced AP-1 transactivation activity and colony formation of JB6 Cl41 cells, following decreased MEK/ERK and JNK/c-Jun signaling. In addition, IL-36γ-induced tumorigenicity of MCF7 cells was significantly decreased in PIN1 KO MCF7 cells. Next, we found that both inhibition of PIN1 by juglone and KO of PIN1 potently reduced the tumorigenic potential of 4T1 metastatic mouse breast cancer cells and in vivo growth of breast tumors to some extent. Hence, our findings demonstrate that IL-36γ-induced epithelial cell transformation and breast carcinogenesis is enhanced by PIN1 via increased activation of the MEK/ERK and JNK/c-Jun signaling pathways. Our results suggest that the IL-36γ/IL-36R axis might serve as a potential therapeutic target in patients with breast cancer.

## 5. Conclusions

In this study, we demonstrated that IL-36γ activates the MEK/ERK and JNK/c-Jun signaling pathways via IL-36R, resulting in the activation of c-Fos, c-Jun, and AP-1 transcription factors in breast cancer cells. PIN1 regulates IL-36γ-induced colony formation in JB6 Cl41, MCF7, and 4T1 cells in vitro. Additionally, PIN1 KO decreased IL-36γ-induced in vivo breast tumor growth to a certain extent. Overall, these data provide new insights into the underlying molecular mechanism of IL-36γ in breast cancer and highlight the novel role of PIN1 in IL-36γ-induced tumorigenesis.

## Data Availability

The data presented in this study are available on request from the corresponding author.

## Supplementary Materials

The following supporting information can be downloaded at: https://www.mdpi.com/article/10.3390/cancers14153654/s1, Figure S1: Full uncropped blots for Figure 2, Figure S2: Full uncropped blots for Figure 3, Figure S3: Full uncropped blots for Figure 4.
