# Supplementary material for: Regulation of Interleukin-36γ/IL-36R Signaling Axis by PIN1 in Epithelial Cell Transformation and Breast Tumorigenesis
Source: Cancers (Basel). 2022 Jul 27;14(15):3654. doi: 10.3390/cancers14153654 (PMC9367291; doi:10.3390/cancers14153654)
Supplement: Supplementary file 1 [file cancers-14-03654-s001.zip › cancers-1791768-supplementary.pdf]

Supplementary Files

Regulation of Interleukin-36γ/IL-36R Signaling Axis by PIN1 in Epithelial Cell Transformation and Breast Tumorigenesis

Muna Poudel, Poshan Yugal Bhattarai, Pratikshya Shrestha and Hong Seok Choi

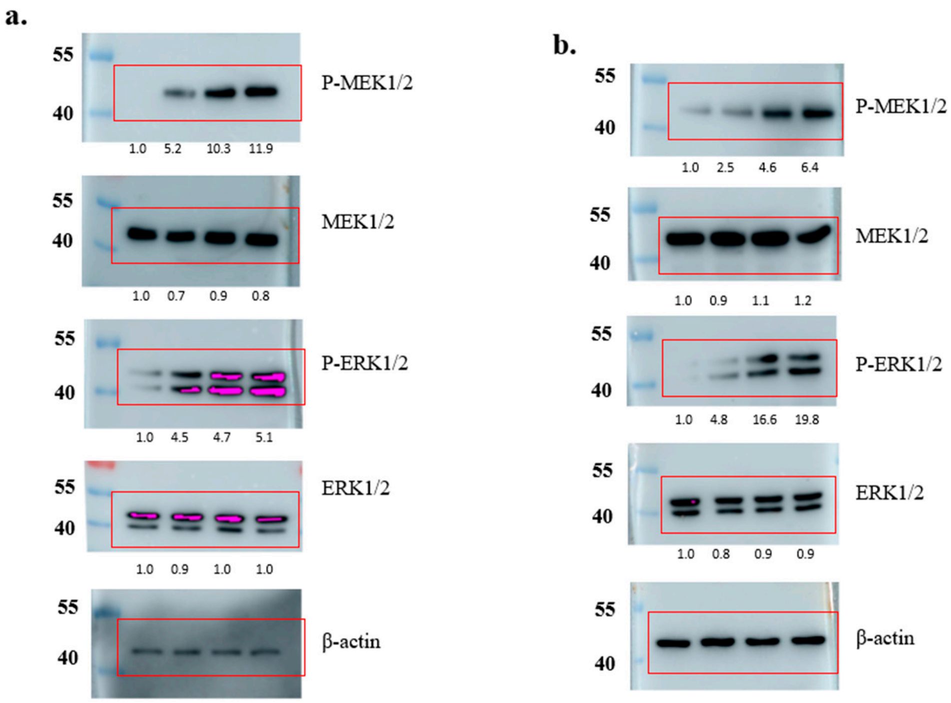

c.

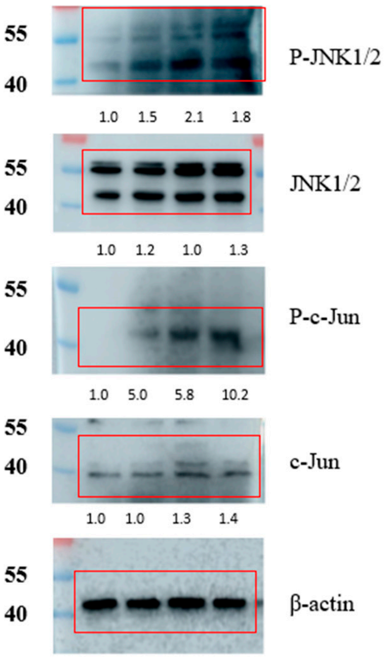
55
40
P-JNK1/2
1.0 1.5 2.1 1.8
55
40
JNK1/2
1.0 1.2 1.0 1.3
55
40
P-c-Jun
1.0 5.0 5.8 10.2
55
40
c-Jun
1.0 1.0 1.3 1.4
55
40
β-actin

d.

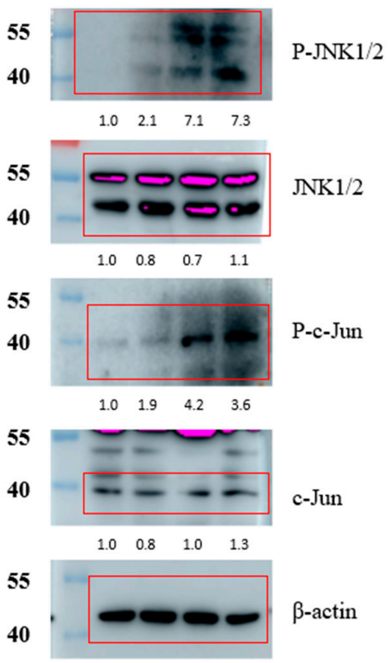
55
40
P-JNK1/2
1.0 2.1 7.1 7.3
55
40
JNK1/2
1.0 0.8 0.7 1.1
55
40
P-c-Jun
1.0 1.9 4.2 3.6
55
40
c-Jun
1.0 0.8 1.0 1.3
55
40
β-actin

e.

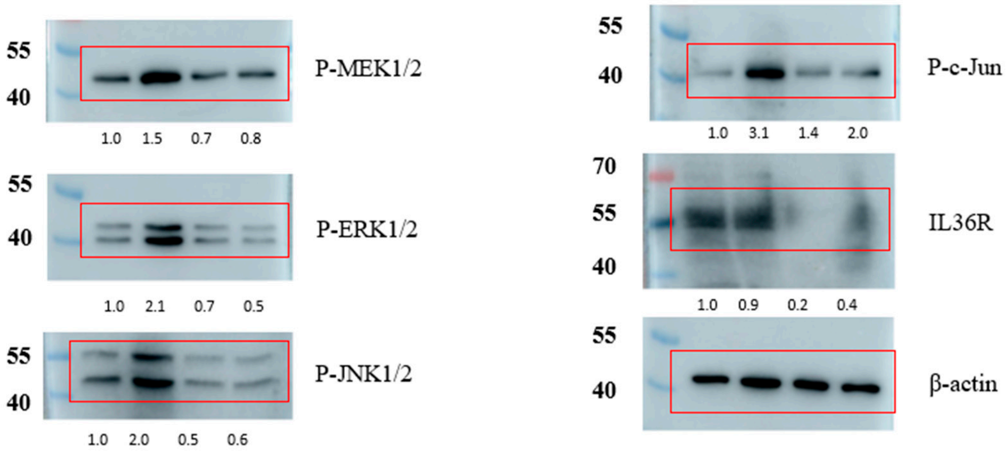
55
40
P-MEK1/2
1.0 1.5 0.7 0.8
55
40
P-ERK1/2
1.0 2.1 0.7 0.5
55
40
P-JNK1/2
1.0 2.0 0.5 0.6
55
40
P-c-Jun
1.0 3.1 1.4 2.0
70
55
40
IL36R
1.0 0.9 0.2 0.4
55
40
β-actin

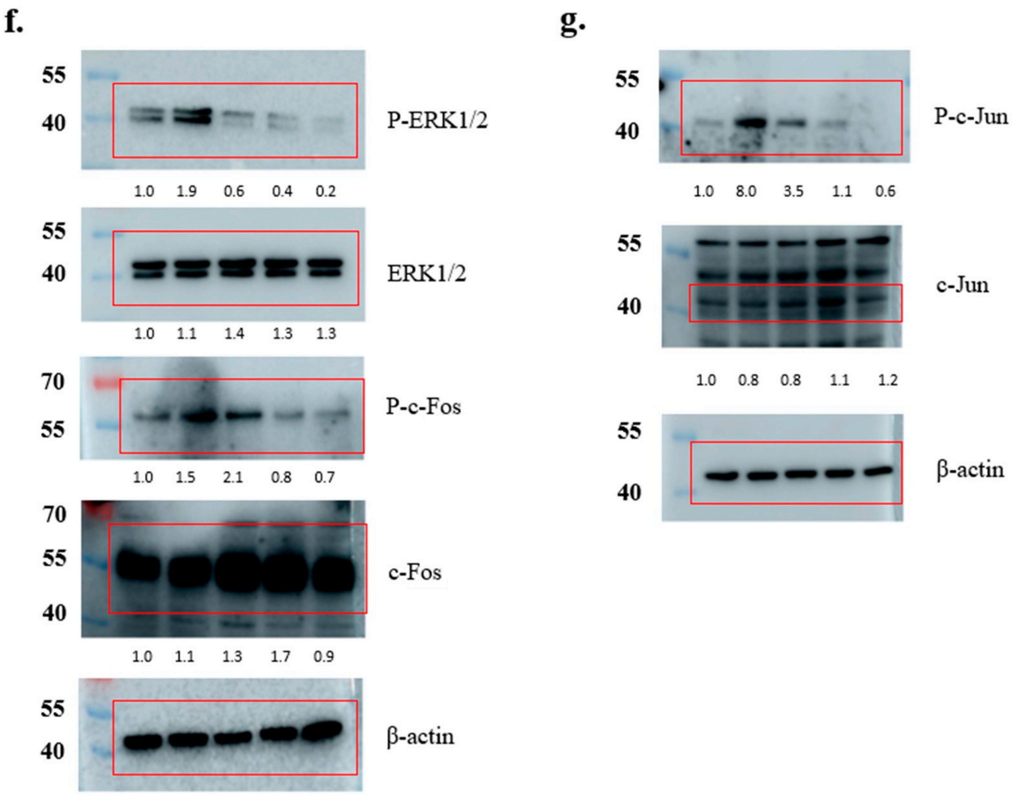

Figure S1. (**a**) Full blots used in Figure 2a; highlighted parts were cropped to make figures. (**b**) Original blots used to make Figure 2b, the portions of blots used in figures are highlighted. The fold changes in the blot intensity of protein were quantified by densitometric scanning using Fiji, normalized to the intensity of the β-actin or total MEK and ERK for each lane, and indicated below the blot image. The quantification was performed on a representative blot image. (**c**) Original blots used in Figure 2c, the parts of blots used in figure are highlighted with in square. (**d**) Full blots used in Figure 2d, highlighted parts were cropped to make figures. The fold changes in the blot intensity of protein were quantified by densitometric scanning using Fiji, normalized to the intensity of the β-actin or total JNK1/2 and c-Jun for each lane, and indicated below the blot image. The quantification was performed on a representative blot image. (**e**) Original blots used in Figure 2e, the highlighted parts were cropped to make figure. The fold changes in the blot intensity of protein were quantified by densitometric scanning using Fiji, normalized to the intensity of the β-actin for each lane, and indicated below the blot image. The quantification was performed on a representative blot image. (**f**) Original blots used in Figure 2f, the parts of blots used in figure are highlighted with in square. (**g**) Full blots used in Figure 2g, highlighted parts were cropped to make figure The fold changes in the blot intensity of protein were quantified by densitometric scanning using Fiji, normalized to the intensity of the β-actin or total ERK1/2, c-Fos, and c-Jun for each lane, and indicated below the blot image. The quantification was performed on a representative blot image.

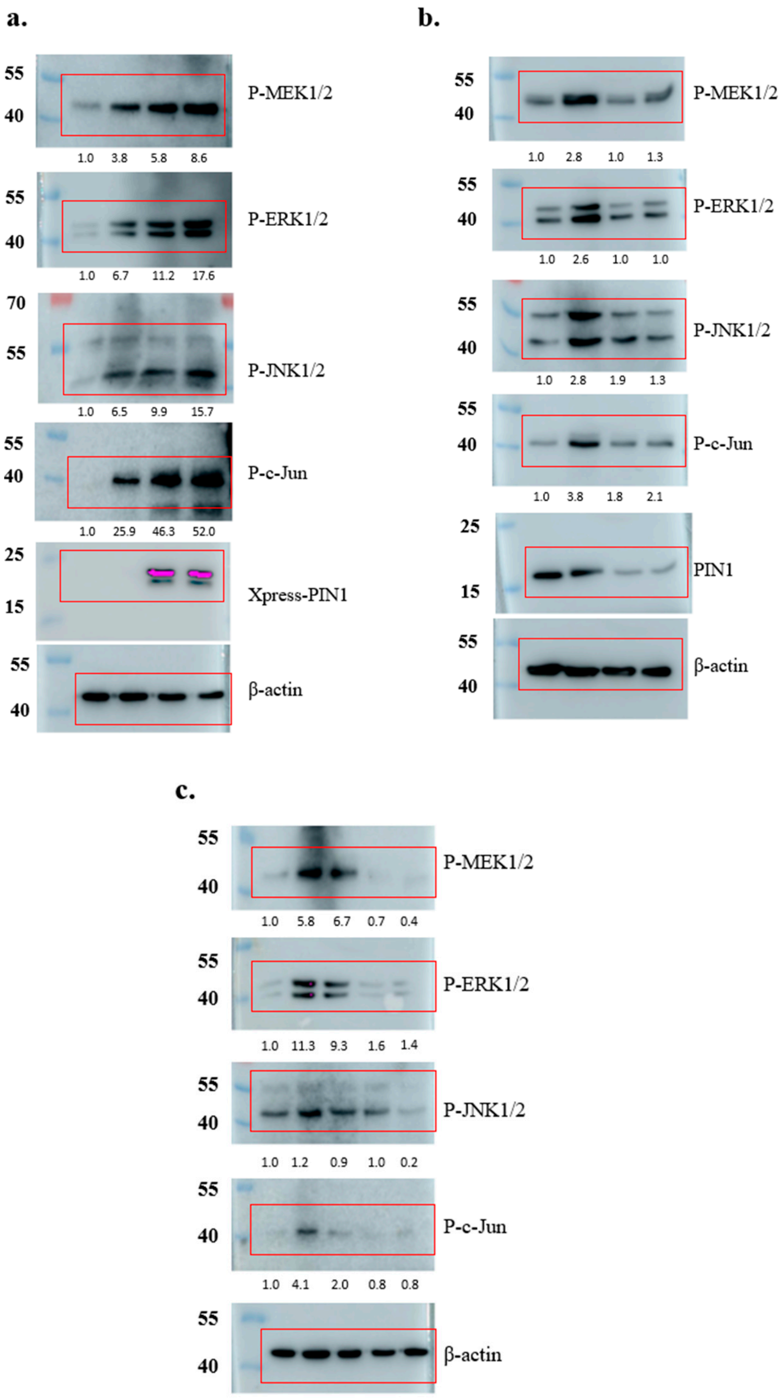

Figure S2. (**a**) Full blots used in Figure 3a, highlighted parts were cropped to make figures. (**b**) Full blots used in Figure 3b, bands used to make figures are highlighted with square. The fold changes in the blot intensity of protein were quantified by densitometric scanning using Fiji, normalized to the intensity of the β-actin for each lane, and indicated below the blot image. The quantification was performed on a representative blot image. (**c**) Full blots used in Figure 3c, highlighted parts were cropped to make final figure. The fold changes in the blot intensity of protein were quantified by densitometric scanning using Fiji, normalized to the intensity of the β-actin for each lane, and indicated below the blot image. The quantification was performed on a representative blot image.

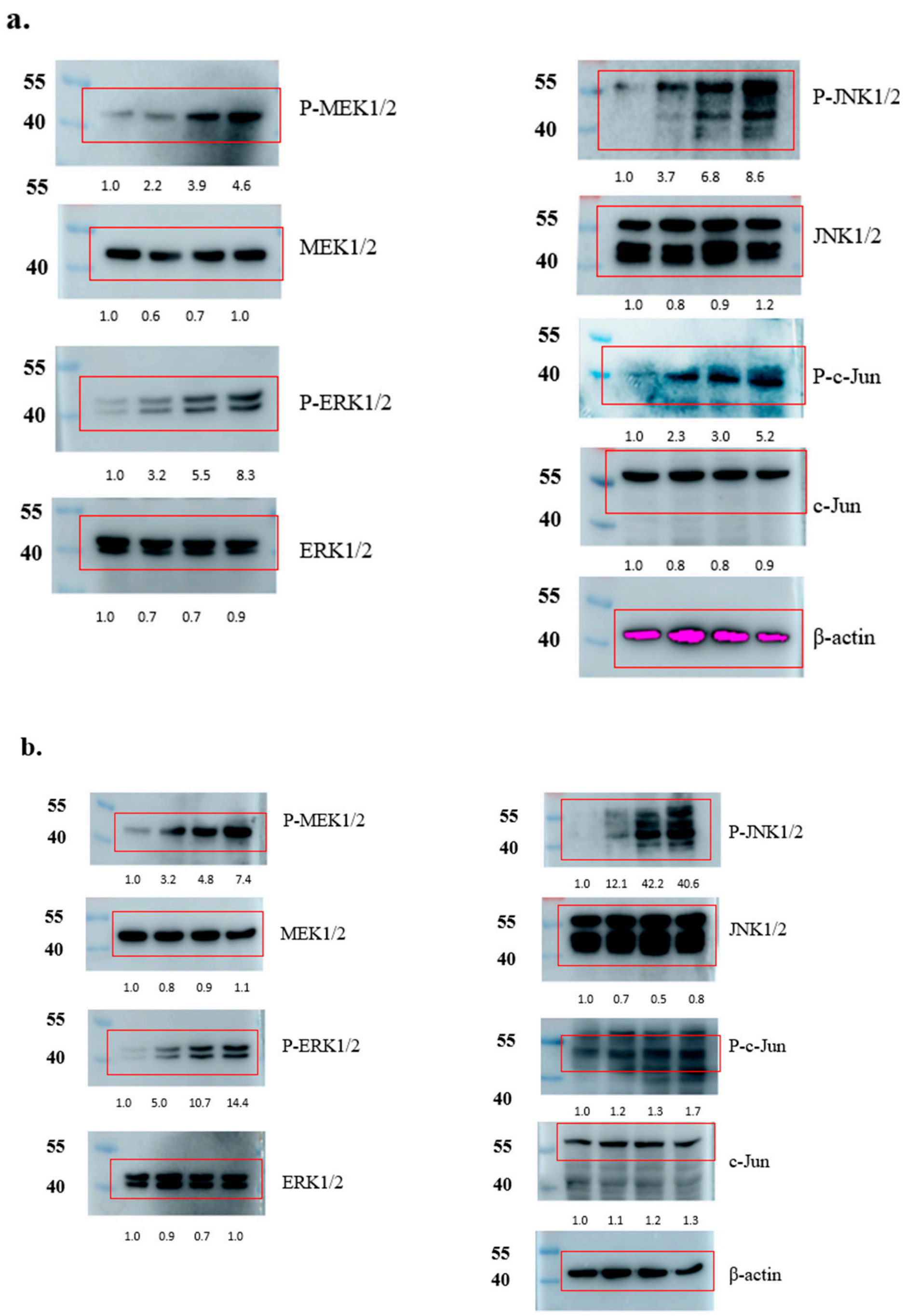
a.
55
40
P-MEK1/2
1.0 2.2 3.9 4.6
MEK1/2
1.0 0.6 0.7 1.0
P-ERK1/2
1.0 3.2 5.5 8.3
ERK1/2
1.0 0.7 0.7 0.9
P-JNK1/2
1.0 3.7 6.8 8.6
JNK1/2
1.0 0.8 0.9 1.2
P-c-Jun
1.0 2.3 3.0 5.2
c-Jun
1.0 0.8 0.8 0.9
β-actin
b.
P-MEK1/2
1.0 3.2 4.8 7.4
MEK1/2
1.0 0.8 0.9 1.1
P-ERK1/2
1.0 5.0 10.7 14.4
ERK1/2
1.0 0.9 0.7 1.0
P-JNK1/2
1.0 12.1 42.2 40.6
JNK1/2
1.0 0.7 0.5 0.8
P-c-Jun
1.0 1.2 1.3 1.7
c-Jun
1.0 1.1 1.2 1.3
β-actin

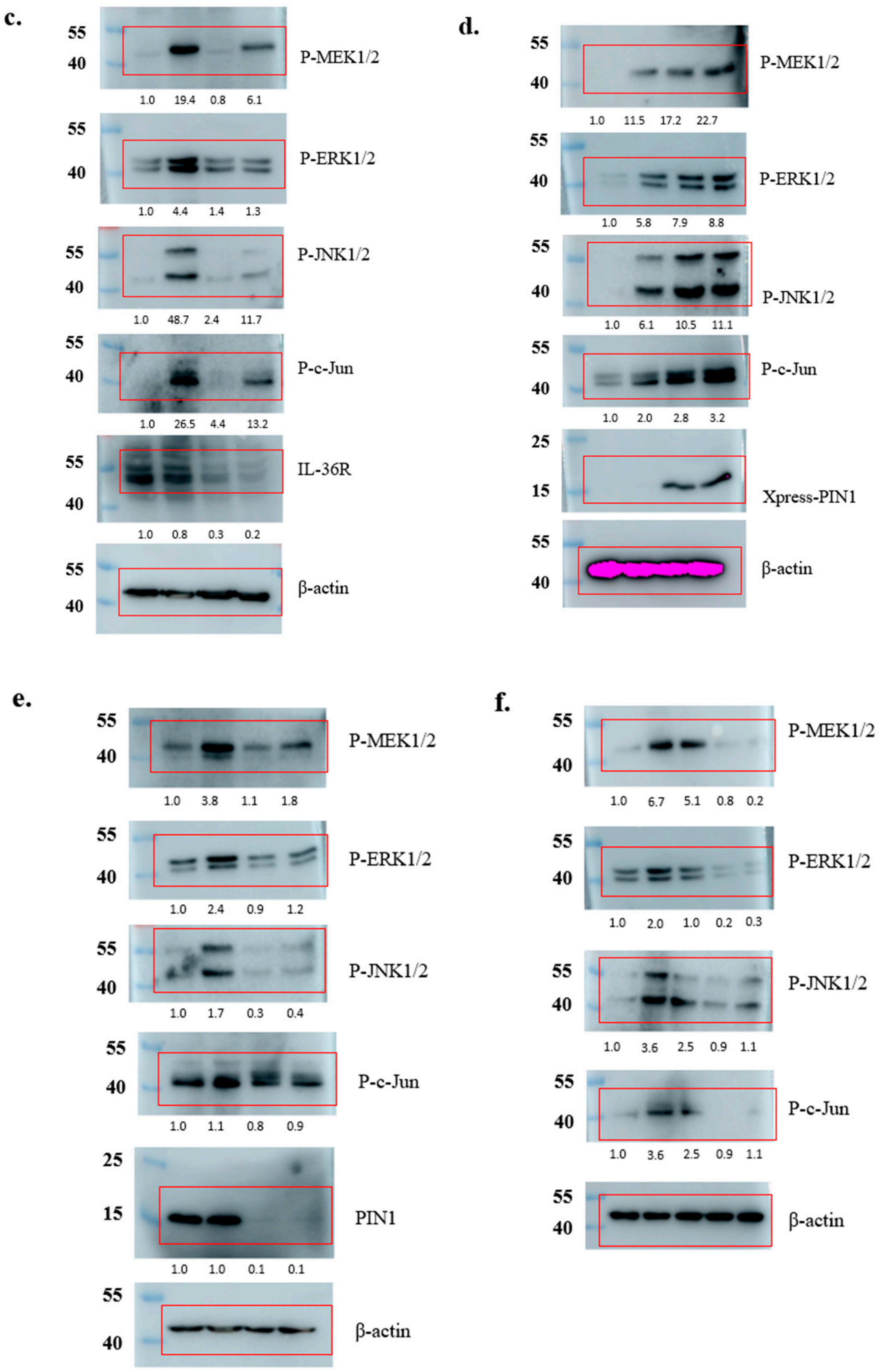

Figure S3. (**a**) Full blots used in Figure 4a, highlighted areas were cropped to make figure. The fold changes in the blot intensity of protein were quantified by densitometric scanning using Fiji, normalized to the intensity of the β-actin or total MEK1/2, ERK1/2, JNK1/2 and c-Jun for each lane, and indicated below the blot image. The quantification was performed on a representative blot image. (**b**) Full blots used in Figure 4b, highlighted areas were cropped to make figure. The fold changes in the blot intensity of protein were quantified by densitometric scanning using Fiji, normalized to the intensity of the β-actin or total MEK1/2, ERK1/2, JNK1/2 and c-Jun for each lane, and indicated below the blot image. The quantification was performed on a representative blot image. (**c**) Full blots used in Figure 4c, highlighted areas were cropped to make figure. (**d**) Full blots used in Figure 4d, highlighted areas were cropped to make figure. The fold changes in the blot intensity of protein were quantified by densitometric scanning using Fiji, normalized to the intensity of the β-actin for

each lane, and indicated below the blot image. The quantification was performed on a representative blot image. (**e**) Full blots used in Figure 4e, highlighted areas were cropped to make figure. (**f**) Full blots used in Figure 4f, highlighted areas were cropped to make figure. The fold changes in the blot intensity of protein were quantified by densitometric scanning using Fiji, normalized to the intensity of the β-actin for each lane, and indicated below the blot image. The quantification was performed on a representative blot image.
